# Applying neurobiology to the treatment of adults with anorexia nervosa

**DOI:** 10.1186/s40337-016-0119-x

**Published:** 2016-12-05

**Authors:** Laura Hill, Stephanie Knatz Peck, Christina E. Wierenga, Walter H. Kaye

**Affiliations:** 1 The Center for Balanced Living, 8001 Ravines Edge Court, Suite 201, Columbus, OH 43235 USA; 2 Department of Psychiatry, University of California San Diego, San Diego, CA USA

## Abstract

**Background:**

Anorexia nervosa is a severe, biologically based brain disorder with significant medical complications. It is critical that new, effective treatments are developed to interrupt the persistent course of the illness due to the medical and psychological sequelae. Several psychosocial, behavioral and pharmacologic interventions have been investigated in adult anorexia nervosa; however, evidence shows that their impact is weak and treatment effects are generally small.

**Method:**

This paper describes a new neurobiological anorexia nervosa model that shifts focus from solely external influences, such as social and family, to include internal influences that integrate genetic and neurobiological contributions, across the age span. The model serves as a theoretical structure for a new, five-day treatment, outlined in this paper, targeting anorexia nervosa temperament, which integrates neurobiological dimensions into evidence-based treatment interventions. The treatment is in two phases. Phase I is a five day, 40 hour treatment for anorexia nervosa adults. Phase II is the follow-up and is currently being developed.

**Results:**

Preliminary qualitative acceptability data on 37 adults with anorexia nervosa and 60 supports (e.g., spouses, parents, aunts, friends, partners, children of anorexia nervosa adults) are promising from Phase I. Clients with anorexia nervosa and their supports report that learning neurobiological facts improved their understanding of the illness and helped equip them with better tools to manage anorexia nervosa traits and symptoms. In addition, nutritional knowledge changed significantly.

**Conclusions:**

This is the first neurobiologically based, five-day treatment for adults with anorexia nervosa and their supports. It is ﻿a new model that outlines underlying genetic and neurobiological contributions to anorexia nervosa that serves as a foundation to treat both traits and symptoms. Preliminary qualitative findings are promising, with both clients and supports reporting that the neurobiological treatment approach helped them better understand the illness, while better conceptualizing how to respond to their traits and manage their symptoms. Data in Phase I shows promise as a neurobiologically based intervention for anorexia nervosa, and it serves as a foundation for the development of Phase II. Evidence of ongoing program efficacy will be described as data are reported on Phase II.

**Trial registration:**

NCT NCT02852538 Registered 1 August 2016.

**Electronic supplementary material:**

The online version of this article (doi:10.1186/s40337-016-0119-x) contains supplementary material, which is available to authorized users.

## Plain english summary

This paper introduces a new, five-day treatment for adults with anorexia nervosa and their supports that integrates neurobiological research with experiential treatment tools. A new neurobiological model is introduced that sets the stage for the possible genetic and brain basis of this deadly illness. In addition, this intervention “treats to the trait," addressing character traits common to anorexia nervosa, such as perfectionism, anxiety and inhibition, while working toward both short and long-term management and change. Nutrition intervention approaches "food as medicine" that "doses" the macronutrients. Pilot results found that there was a significant improvement in nutritional knowledge between the beginning and end of the week. Over 95 % of the clients and supports reported they enjoyed learning about the neurobiology of the illness and that they found the experiential activities improved their understanding of anorexia nervosa. Over 95 % of the clients reported that they felt their family/friends were equipped with better support tools and over 90 % of the supports and clients reported the treatment both met or exceeded their expectations. Ideas on how this short-term intense intervention could be integrated into ongoing eating disorder treatment are explored.

## Background

Anorexia nervosa is a severe, biologically based brain disorder with significant medical risks [1] and a tenacious development over time. While onset is common during puberty and adolescence, it also occurs throughout adulthood [2, 3]. Up to 50 % of individuals with anorexia nervosa develop a chronic course, characterized by significant physical and psychological impairment [4–8]. When the illness is active for five to seven years or more, it becomes chronic and enduring [9]. The longer a person has anorexia nervosa, the harder it is to overcome [10].

The development of effective treatments is of critical importance to interrupt the persistent course of illness, given the magnitude of medical and psychological sequelae. Brain imaging research has led to an increased understanding of the etiology and neurobiological mechanisms underlying anorexia nervosa symptoms and has identified potential targets of treatment, such as the ventral striatal area. Despite this new perspective, there are no interventions integrating the current, empirically supported, biological findings into treatment.

Several psychosocial, behavioral and pharmacologic interventions have been investigated in adult anorexia nervosa; [4, 5, 11–15] however, evidence supporting currently available treatments is weak, and treatment effects, when found, are generally small [15–22]. Treatment for adolescent anorexia nervosa is more promising, with a large evidence base supporting family-based treatment (FBT) [23–26]. Although the level of parental control prescribed in FBT is not well-suited for adult individuals, elements of FBT may be beneficial for adult treatments. Finding creative alternatives to integrate support persons as agents of change becomes the challenge for adults who are more autonomous and often have little to no support networks.

A new model that highlights the mechanistic pathways, constructs and traits underlying anorexia nervosa psychopathology as one ages, as opposed to focusing solely on a symptom-oriented approach, is in accord with the National Institutes of Health (NIH) recommended Research Domain Classification (RDoC). Such a model could conceptualize a bio-genetic etiological foundation for anorexia nervosa, to inform and guide treatment approaches. For instance, white matter prefrontal cortex neural growth occurs until early thirties [27]. Treatment for adults needs to address how the brain can change through neuroplasticity, and to﻿ ﻿explain that this process takes longer compared to adolescence. If adults with anorexia nervosa learn about neurobiological details that contribute to the neuropathway alterations in a method that matches their manner of thinking, in detail, then they may be open to accept the need for more time and additional support to compensate for what their body/brain cannot do, in spite of their desire to overcome the illness alone.

This paper introduces a new anorexia nervosa model that integrates genetic and neurobiological influences in relation to social/external pressures for thinness across the age span, and then introduces a new anorexia nervosa adult treatment approach with supports (e.g., family, friends) that experientially integrates anorexia nervosa temperament with neurobiological research findings in a five-day intensive treatment. The treatment targets adults with anorexia nervosa or those who have a history of anorexia nervosa and share common traits.

## Philosophy on the use of neurobiology in anorexia nervosa treatment

In order to make advances in adult anorexia nervosa treatment, a paradigm shift is needed that includes consideration of both the biological and the familial dimensions for adult anorexia nervosa treatment. Targeting anorexia nervosa symptoms alone has not proven effective. Therefore, targeting underlying neurobiological mechanisms, that are core expressions of anorexia nervosa traits, might show promise. It is hypothesized that traits such as perfectionism, harm avoidance, anxiety and inhibition manage personality and behavioral responses are present prior to the development of anorexia nervosa and continue long after recovery [28–30]. These traits may be core maintaining or perpetuating factors impeding treatment engagement and may be important in helping those who have long-standing, or severe and enduring anorexia nervosa.

Additionally, persons with anorexia nervosa traits tend to focus on details and are less capable of seeing the global picture [31, 32]. For example, a child who has obsessive and perfectionistic traits may repeatedly focus on details in school assignments and find her/himself overwhelmed with a multitude of choices. Uncertainty sets in, as concern about making the “right” decisions increases, as traits establish a stronger influence during adolescence.^[77]^ Those very traits could be sublimated to help the adolescent and adult direct attention from thoughts that appear to “freeze” or get “stuck,” from too many options directed at them at the same time, to thinking freely through selections when given fewer options. This approach could transform a problem into a solution.

Similarly, the ability to make and trust decisions is critical for adolescents and adults to function autonomously in everyday life situations. The inability to make and trust decisions is reported by many adult anorexia nervosa clients in our treatment programs. Trust in one’s decisions is diminished when a reduction in dopamine occurs in the ventral limbic area of the brain [33–35]. If, through treatment, clients learned the neurobiological contribution to their inability to sense and make decisions, they might be empowered to shift their understanding from self-blame to focus on what is needed to compensate for brain misfiring.

In addition, explaining the biological basis of anorexia nervosa may help clients shift fault from the difficulty and inability to eat with ease, and reframe it to a biological response that impacts their eating and decision making ability. Planning and structure can create a method to augment difficulty in decision making.

## A neurobiological model for anorexia nervosa

There have been significant advances in our understanding of etiological influences associated with anorexia nervosa. Genetic studies indicate that heritability accounts for approximately 50–80 % of the risk of developing an eating disorder (ED) [36]. Recent imaging studies reveal that individuals with anorexia nervosa tend to have common temperament and personality traits related to neural circuit function, which are heavily implicated in the development and maintenance of the disorder [31, 34, 37–42]. The temperaments are characterized by increased trait anxiety [43] and state anxiety related to food and eating, [44] high incidence of co-morbid anxiety disorders, [43] high punishment sensitivity and low reward reactivity, [45] elevated intolerance of uncertainty [46] and exaggerated harm avoidance (HA). HA is a multifaceted temperament trait [47] that contains elements of anxiety, inhibition and inflexibility [28–30, 48, 49]. There are multiple ways in which these traits may influence the etiology, maintenance and treatment of the disorder. For example, elevated anxiety and HA not only maintain ED symptoms, but may predict poor treatment outcome; [28, 50–54] and higher levels of pre-meal anxiety predict lower food intake [44]. This is offset through the anxiolytic effect from acute dietary restraint and caloric restriction [44, 55]. Food consumption stimulates a dysphoric mood, [56] suggesting that anorexia nervosa individuals may regulate eating behavior to manage anxiety.

**Fig. 1:**
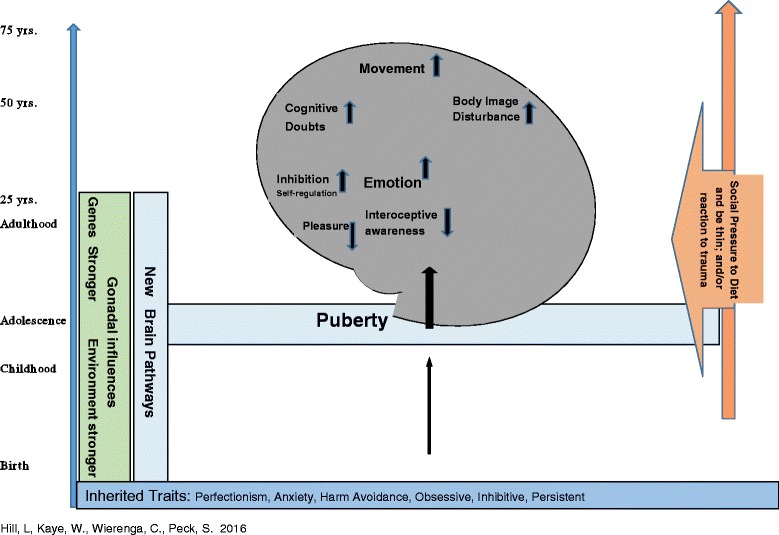
A Temperament-Based Model for Anorexia Nervosa

A new anorexia nervosa model is proposed that reflects temperament and alterations in brain circuitry in order to inform and help guide treatment interventions (Fig. 1). This anorexia nervosa model shows heritable traits, such as harm avoidance, perfectionism, anxiety and inhibition, establishing a temperamental basis from which the illness may develop [28–30, 48, 49]. These traits are initially expressed behaviorally in childhood. Common temperamental expressions may include a natural compliance to rules within the family/school and social settings. Likewise, perfectionism may be expressed through high grades and excellent performance, and it may be perceived as never good enough and experienced with little reward sensation. Alterations in reward and punishment reactivity also account for the tendency for anorexia nervosa individuals to see their own errors over their successes. Individuals with anorexia nervosa often demonstrate a natural preference towards highly structured and predictable environments due to difficulties with tolerating uncertainty and set-shifting. As such, anxiety appears to reduce when children, adolescents and adults with anorexia nervosa are provided with structure verses open-ended and more ambiguous tasks. These same traits also influence response to food for individuals with anorexia nervosa. Individuals with anorexia nervosa tend to report that they do not experience a sense of reward in response to food intake, but instead experience anxiety. As such, restriction of food intake appears to provide an anxiolytic effect [57].

**Fig. 2:**
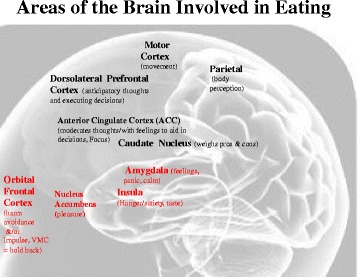
Areas of the Brain Involved in Eating

Recent research, including our own, has implicated neural substrates in the ventral (lower or bottom area) limbic circuitry, dorsal (top) cognitive circuitry and insula, underlying altered reward processing [37, 58, 59], cognitive or self-regulatory control [32, 60–62] and interoception [41, 63–65] in the pathophysiology of anorexia nervosa (Fig. 2). The ventral area of the limbic neural circuit includes the nucleus accumbens, putamen and caudate, as well as the orbitofrontal cortex and amygdala. These regions code for the rewarding and motivating value of eating and contribute to approach or avoidance behaviors.

The dorsal cognitive network makes and executes decisions, such as to control food consumption, based on considerations of both short- and long-term outcomes (e.g., perceived weight gain). It includes the dorsal caudate and dorsal anterior cingulate, lateral prefrontal cortex and parietal cortex. Specifically, the insula is a hub for the evaluation of interoceptive cues, such as internal pain, tastes or feelings of fullness, and plays a pivotal role in anticipation and processing of interoceptive states by conveying information about the internal milieu of the person or organism [66, 67] and perceived importance or salience of a food stimulus. These systems interactively weigh the reward value of food and consequences of consuming it, and integrate this information with homeostatic and motivational drives to guide eating behavior.

The model describes brain response changes for those with anorexia nervosa from mounting evidence that indicates that an altered balance of reward and inhibition may contribute to disordered eating [1]. Figure 1 demonstrates that in patients with anorexia nervosa, severely restricted food intake appears to be related to overactive inhibitory control (up inhibition arrow) in combination with underactive reward circuitry (down interoception and pleasure arrows). Imaging data further suggest that anticipatory anxiety contributes to restricted eating. For example, individuals with anorexia nervosa report exaggerated anxious and avoidance responses to food cues [41, 44]. This is reflected in ventral striatal limbic responses (up emotion arrow) [41] and implicates elevated HA and anxiety in anorexia nervosa. This may be expressed and experienced as cognitions becoming a cacophony of “noise” impacting the inability to decide or execute decisions, thus increasing doubt in the dorsal caudate (up arrow). This also entails altered dopamine (DA) and serotonin (5-HT) function. This disconnect between anticipating and experiencing food stimuli likely contributes to restricted eating in AN. Lowered dopamine and increased serotonin response may also contribute to the ability of persons with anorexia nervosa to delay rewards since reward may be experienced as less pleasurable [41].

Evidence also suggests parietal disturbance [68], which codes for perception of body image and shape disturbance increases (up body disturbance arrow); and the motor cortex area implements the overactive anxiety and body shape disturbance through excessive exercise (up movement arrow). Together, these findings have implications for motivation to eat and ability to evaluate reward and make decisions. It is plausible that elevated harm avoidance, perfectionism, anxiety and inhibition, present during childhood, may increase the inclination of compliance with rules for adults with anorexia nervosa.

The model shows that nurture, or environmental influences, have more influence on the child’s temperament pre-puberty compared to post-puberty [58]. The hypothalamic-pituitary gonadal axis initiates biological changes during puberty, contributing to a shift from nurture to nature taking the dominate role in trait expression through mid-twenties [69]. This may trigger changes in neurochemical circuity and contribute to how thought patterns, emotions and motor expression alter [70, 71]. In our view, to acknowledge that anorexia nervosa tends to develop around puberty and may rise in incidence again around 18, when faced with a multitude of new life and daily decisions, is not enough to understand the illness. In addition, the arrow on the right, social and traumatic experiences, influence and may even alter genetic expression during adolescent and adult life, contributing to the development of anorexia nervosa (shown as the arrow on the right in the Figure).

Neurobiological findings indicate and direct us to possible truths. However, research holds little value if clinicians and clients do not understand it or know how to interpret it. Neurobiologically based research is new to the treatment field and needs to be presented to anorexia nervosa adults and their supports in a way that they can understand the findings, so the client can identify and determine how the findings relate to their own experience. Interpreting research accurately and creatively in a manner that enhances understanding can lead to increased motivation, instead of resistance, to change.

To date, anorexia nervosa clinical treatment approaches have not been developed and/or updated to include both current neurobiological research findings and family-based approaches for adults. The most widely used behavioral treatment models, such as cognitive behavioral therapy (CBT or CBT-Enhanced), focus on symptomology. Biological underpinnings of the cognitions and behaviors are given less to no focus.

Hence, what does a neurobiologically based adult anorexia nervosa treatment look like?

## Method: Overview of the NEW FED TR treatment

NEW FED TR is a five-day treatment for adults with anorexia nervosa. It is a neurobiologically informed, interactive, family-based treatment that draws upon the specific etiological traits characterizing anorexia nervosa [72]. It stands for Neurobiological research findings Enhanced With Family/Friends of those with Eating Disorders, addressing Trait Responses that both create a vulnerability to the illness and serve as markers for strength and recovery through treatment (formerly reported as Temperament-Based Treatment). NEW FED TR is being developed in two phases.

Phase I is for females or males who are 18 years old and above (the program is expanding to 16 years old and above), who have a history of anorexia nervosa or current diagnosis of anorexia nervosa. Persons with a current diagnosis of bulimia nervosa (BN) or binge eating disorder (BED) and have a history of anorexia nervosa may be in the treatment program if their temperament is comprised of anorexia nervosa traits. NEW FED TR requires at least one support person (e.g., parent, spouse, partner or friend) and allows up to four supports per client. The client and supports come together to learn about anorexia nervosa together with a biological perspective and to explore why and how the supports are needed.

It is an experiential treatment where all neurobiological information is applied and integrated through activities that provide a clear reliable structure that set a foundation for safety while learning and practicing new approaches to manage the traits and symptoms. Information is presented in the manner that anorexia nervosa clients tend to think: in detail, not in generalized points. Clients are treated as the “experts” who know their illness well, and thus have much to offer when specific questions are asked by clinicians on how the neurobiological research findings are experienced or apply to them. This appears to transform resistance into client motivation.

Phase II is the follow-up. It is being developed currently by gathering input monthly from the clients and supports who have completed Phase I. They identify what is additionally needed for this phase and report how they are eating and using the tools learned while functioning in their home or other treatment settings.

How to best integrate Phase I into ongoing treatment, or offer it as a free-standing program, is currently being studied to determine best impact. Phase I has been developed for those who meet criteria for partial hospital program (PHP), intensive outpatient program (IOP) or outpatient (OP) levels of care.

The development of Phase I has been over the last four years and has been in preliminary testing over the last two years. Client and support feedback have been the foundation to refine and develop the client/support manual, group sessions and neurobiological tools. The suggestions for changes have evolved from many recommendations for alterations to consistently requesting that the schedule, activities, tools and manual remain the same.

The clinical reliability has been established over the past two years via open treatment trials, allowing the treatment team to refine the schedule and neurobiological content, approach to meals, movement and Behavioral Agreement until they have been consistently reported by clients and supports as above average to excellent as presented. Similarly, the average rating for each hour of the treatment program, instruments and client/support manual is “no changes recommended.”

The Behavioral Agreement is a 16 page treatment plan written for and with the clients, supports and therapists to establish a clear structured plan in response to primary anorexia nervosa traits and symptoms (Sample is found as Additional file 1). It provides clients and supports with structure while addressing individual concerns. It is a central tool that identifies how each client chooses to manage their traits, tools and decisions when at home. It also serves as a transitional treatment tool when leaving NEW FED TR Phase I and entering Phase II.

An intent of Phase I is to provide detailed nutritional information, neurobiologically based information and tools that structure while drawing upon the ideas and feedback from the supports and experts (clients). It is the client and support who apply and practice the practical treatment methods to better manage anorexia nervosa traits and symptoms at home or in ongoing treatment. The supports hear the same information and learn the same tools in tandem with the adult clients. Their presence allows them to learn and practice the tools at the same time as the clients.

To date, Phase I has been integrated or served as a “jump-start” or “boost” at the beginning, middle or end of an ongoing PHP, IOP or outpatient (OP) treatment, based on client symptom severity. NEW FED TR has also served as a stand-alone treatment because no other eating disorder treatment was available. In these cases, supports can be significant agents of change. They often assume a greater role in ongoing assistance to help shift the anorexia nervosa traits toward constructive expressions over time. They also realize the importance of increasing structure, planning, rules and rituals into daily patterns. Follow-up data will help determine the impact of Phase I in these multiple roles.

NEW FED TR has accepted clients with body mass indexes (BMI) ranging from 13.81–30.4. It is recommended that the five-day treatment, Phase I, be followed by additional PHP, IOP or OP treatment depending on level of severity. Ongoing monitoring of food intake, body composition/weight and symptoms, as well as facilitation of continued use of clinical tools, is essential. The supports are appearing to be a central source in helping the anorexia nervosa client transition with continuity into the home setting, even if the supports live in different locations.

Clinicians who assume the client for treatment at their home sites are asked to adopt the Behavioral Agreement established during NEW FED TR and integrate the details that are clearly laid out by the client and supports. This has had varying responses from different clinicians ranging from openly welcoming the treatment tools and Behavioral Agreement and including family and supports in ongoing treatment to rejecting and ignoring the Behavioral Agreement and supports in their practice. It has also been stated repeatedly by anorexia nervosa clients leaving NEW FED TR that the clinicians at their home sites know little to no anorexia nervosa neurobiological information to help them better manage the illness.

A NEW FED TR therapist manual is near completion. It outlines and details every point made during NEW FED TR to aid in consistency across treatment sites. In addition, there is a completed client and support NEW FED TR manual that has been endorsed from client and support feedback. It is used throughout each day of Phase I and taken home at the end of the five-day treatment.

## NEW FED TR phase 1: Five-day treatment outline

**Fig. 3:**
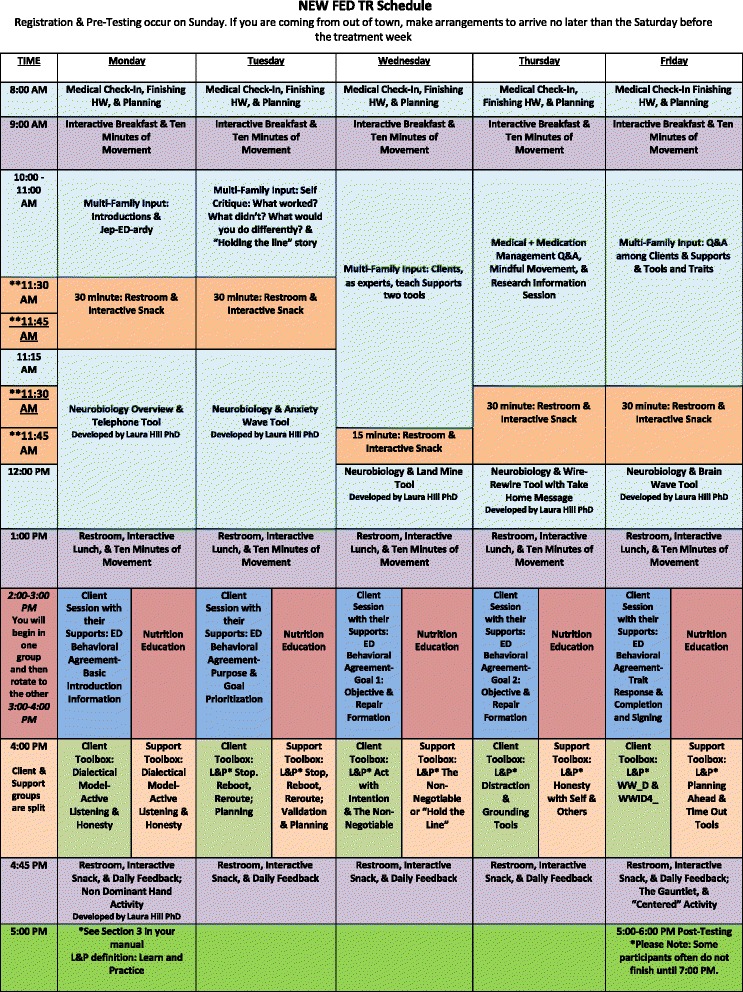
Schedule

NEW FED TR consists of five daily modules aimed at targeting anorexia nervosa trait constructs, delivered with clients, supports and treatment providers. Each module is formatted to deliver: 1) interactive, neurobiological psychoeducation; 2) tools used within a neurobiological context to compensate for brain deficits and facilitate constructive methods for redirecting temperament expression; 3) support management strategies; 4) experiential learning focused on practicing the implementation of the tools that can become skills; and 5) an approach to food as medicine by “dosing” the macronutrients. See Fig. 3 to review the NEW FED TR schedule by modules. The details to the modules and how they are structured in treatment are described in the therapist manual, which is in the final stages of being written.

Every hour of each of the five days the clients, supports and therapy team work together to learn, experience, practice and establish a practical, livable structure for food and daily life activities at home. The only exception is Module 3, where the clients and supports enter separate groups to learn and practice new tools among peers. Clients empathically explore their thoughts, feelings and behaviors together while learning new therapeutic tools.﻿ Simultaneously, supports learn and apply new tools with one another. They draw upon group members' support and understanding regarding difficult and complex life situations.

## Role of the treatment team and census

The comprehensive treatment team is comprised of the client; the clients’ family, friends, partners and others who serve as primary supports; and clinical eating disorder therapists, dietitians and medical staff. Adults with anorexia nervosa are viewed as the experts.

At least one support (the same person) is required to be present every hour of the 40 hours, so that all participants are hearing the information and ideas together and everyone is participating in practicing what each member can do to contribute to change. Clinicians from the clients’ home sites are allowed to participate as a support or team member. This enhances treatment consistency. The intensive nature of Phase I is intended to immerse the client and supports with experiential learning, apply and practice tools every hour, practice food preparation and use movement or “walk-abouts” after meals with a trained therapy team interpreting key factors involved in brain and body recovery.

 Adults with anorexia nervosa tend to want to “go it alone,” avoid help or are hesitant to ask for support from those they know. They often report fearing that their spouses, parents or friends will “hover too much” or take control of their lives. Keeping the supports from participating encourages the anorexia nervosa fears and prevents supports from learning what they can do, and not do, to help their loved ones. Supports are not only a form of intervention and growth during and after treatment, they become agents of change, helping the adult with anorexia nervosa enter treatment.

The treatment is comprised of up to six anorexia nervosa adult clients with up to four supports per client working together in a multi-support group format. This totals up to 24 clients and supports with three clinicians, one dietitian and one medical staff person, plus nutritional and administrative assistants. The group format allows both clients and supports to see similar qualities in one another that help validate feelings and encourage motivation for change while including the client and supports as experts who have lived with the illness and know it from many angles. It assumes the total team holds creative answers for solutions while the program models, throughout the treatment week, also take a team to “manually” change adult anorexia nervosa traits and symptoms until possible future neurobiological advances can be made.

## Role of neurobiology and temperament in treatment

NEW FED TR “treats to the traits” throughout each day via experiential, neurobiologically based activities and playful clinical tools. The mental, and at times physical, pain from eating is acknowledged and used experientially and made malleable by transforming it through play. The treatment approach acknowledges that underlying traits are persistent through life (versus transitory), and focuses on teaching adults with anorexia nervosa and their supports to manage anorexia nervosa symptoms by using their traits constructively (versus destructively through the pursuit of emaciation). One NEW FED TR client shared, “Anorexic individuals, who may be men or women, are both terrified of and obsessed with food…Therefore, the Center has resolved to treat food as medicine. Our meal plans are ‘prescriptions’ and each of us must take a certain number of ‘doses’ of each macronutrient per day…The irony is that this program teaches anorexics to use the exact method that led us into our illness to bring us out of it. All the planning, calculating and perfecting that was once used to chip away at ourselves can also be used to achieve optimal health.”

**Fig. 4:**
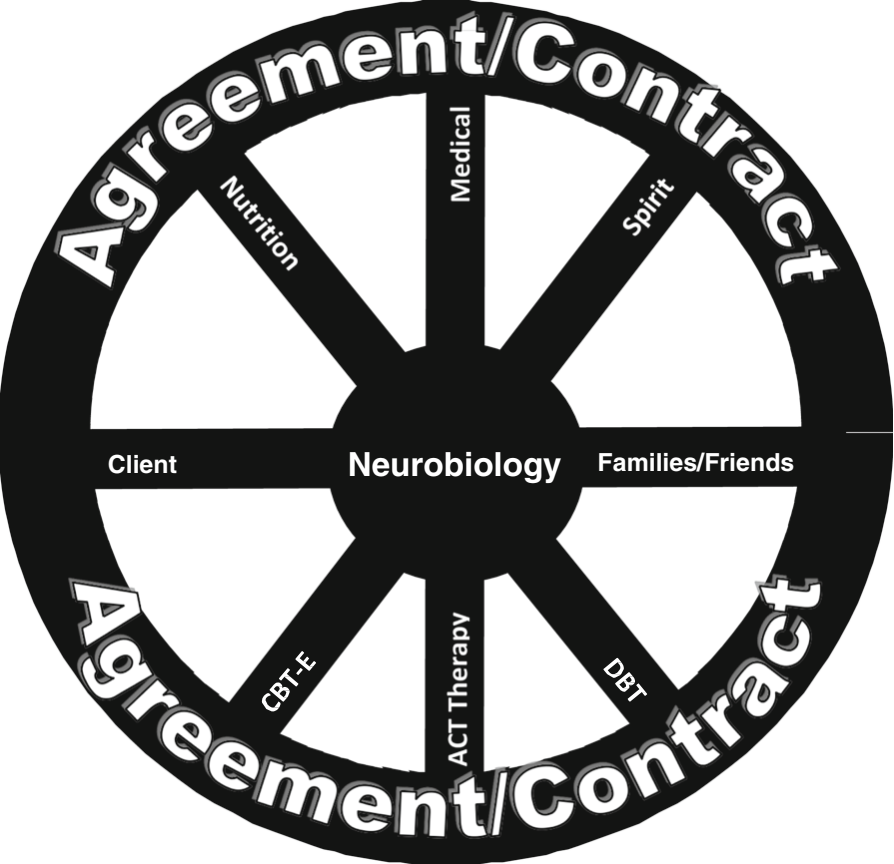
How Neurobiological Findings Relate to Other Treatment Tools and the Behavioral Agreement

Neurobiological research facts are explained and integrated into a group family-based therapy (FBT) format for adults, synthesizing evidence-based dialectical behavioral therapy (DBT) and cognitive behavioral therapy (CBT) tools into experiential activities that have been tested with clients and supports. Figure 4 visually describes how neurobiological information is approached in NEW FED TR in relation to other forms of treatment, support persons and the Behavioral Agreement. The treatment approaches neurobiology as the hub of a wheel, out of which CBT, DBT, and other treatment brain-based tools can be applied. The neurobiological findings helps explain why the clients need to use the treatment tools, and the role supports, medication and the Behavioral Agreement play, to hold the structure and trait management in place. The therapy team approaches the clients and supports from a perspective of “I need your help to figure out if this applies to you.” Asking their input on what their experiences are like allows for the clients’ voices to be heard in a context of the expert.

In addition, real-time client and support interactions during target events such as mealtimes, acute emotional distress, behavioral resistance and family interactions allow for both clients and supports to receive applicative, hands-on experience to refine management skills. The intensity of intervention is designed to capitalize on tenets of neuroplasticity that require increased consistency and clear structure as critical components needed to elicit behavior change for anorexia nervosa [73, 74].

A Behavioral Agreement (Additional file 1) instrument has been developed and validated that integrates neurobiological concepts and findings into a detailed structure to help each client decide what s/he wants to change the most and what actions are most and least helpful to make that change for themselves, the supports and for other therapy, medical and nutritional team members. Client feedback has refined it to the place that the majority report “I would not change a word, this is what I think is true for me.” It is more detailed and structured than other treatment approaches. For example, it offers multiple choices instead of open ended questions that address the identified and prioritized goals.

## Neurobiologically based tools (Module 2)

The clients discover through neurobiologically based experiential tools that improving their health means there is increased struggle mentally, interpersonally and perceptually, not less. While other diagnoses, such as depression and anxiety, bring greater peace and calm as the person improves, anorexia nervosa is the opposite. More distress is experienced the more one eats to improve their physical health state. This struggle is explained through several different experiential activities, such as the Non-Dominant Hand [75].

The clients and supports participate in a guided activity writing different phrases with their non-dominant hands. All clients and supports are asked to share what they feel as they write with their non-dominant hand. Common responses include: awkward, stupid, slow, incapable, irritated, inept and frustrated. The analogy is made that eating, for those with anorexia nervosa, is a non-dominant action and response. During the activity, the clients consistently validate that the eating experience encompasses the very feelings the supports reported when writing with their non-dominant hand. The supports report realizing the difficulty that their loved ones feel when eating what appears to be “normal foods” and “normal amounts of food.”

Supports are asked what they would do if they were told they had to write with the non-dominant hand the rest of their life. Many report they would stop writing, and the analogy is then realized regarding the day after day struggle their loved ones experience when eating. Clients with anorexia nervosa report they do not feel better when eating what is needed for their bodies, they feel worse, and that this activity helps them to help the supports better understand and begin to explore what actions are needed to cope with and manually help the clients to eat. Practice becomes the identified and proverbial key to tolerate the foods. The clients are expected to eat, even though it is a “non-dominant” brain response, by eating enough food for the rest of their lives.

As one client wrote on what helped the most that day in treatment, “The experiential exercise. So important for support people to get a taste of what people with anorexia go through on a daily basis.” A support person wrote, “[The] illustration of the difficulty experienced by my daughter - in how learning what she is feeling and thinking - her response and those of others when it came to the surface in the group discussion.”

This illness of anorexia nervosa is described as learning to “take their medicine,” which is food. Food has “side effects” in the brain. Thus at times, chemical medications are needed to treat the “side effects” of their primary medication, food. Hence, anorexia nervosa appears to be an illness that induces a complex struggle to progress toward health.

Other neurobiologically based activities that have been tested and refined from client and support feedback over the last five years include: Telephone, Anxiety Wave, The Landmine, Wire Rewire, The Brain Wave [75] and The Gauntlet.

## Nutritional intake: Food is medicine and macronutrients are “dosed” (Module 5)

The clients engage in a nutritional assessment prior to entering the program. Their body weight/composition serves as a foundation in establishing their meal plans, along with their value/philosophy around food. For example, if they report being vegetarian or vegan, that was honored and their meal plans incorporated that approach. The approach to food in NEW FED TR is that food is medicine and the macronutrients are “dosed.” The message is that since eating disorders are biologically based, then approaching food as medicine is similar to approaching insulin in doses as one does with diabetes. The meal plan becomes the “prescribed macronutrient combinations and amounts” that are matched to the client’s activity and body composition with the aim to establish a healthier body for the identified life activities established in the Behavioral Agreement [76].

Each client prepares their own meals and selects their own snacks each day of the treatment, with their supports learning and assisting beside them. Supports also prepare their own meals. The clients prepare and apply the macronutrient “doses” they are “prescribed” by the dietitian and supports learn and then practice each meal on how the meal reflects the dosed amounts. For example, a breakfast may be a “3,2,1” dose, being three carbohydrates, two proteins and one fat. Fats are described in the program as “endurance fuels” or “EFs” [76]﻿. The program ﻿aims to provide ﻿foods similar to what the clients can purchase and use at their home sites to help with transition from treatment to home. As one client reported in the end of the week feedback, “I loved that I had the ability to choose the foods that fit my meal plan. It was more realistic to have normal grocery store food to simulate what it would be like at home.”

A Nutritional Facts Questionnaire was developed by the eating disorder dietary team who were involved in the program development. The nutritional facts used in the questionnaire were based on macronutrients “dosed” in units, drawing from the American Dietetic Association Exchange Lists for meal planning (See Additional file 2 to review the questionnaire and scoring instructions). Both clients and supports are tested on nutritional facts applied in the treatment to assure the same information was learned and practiced at home by both parties and to encourage consistency. By dosing foods with supports’ oversight, there may be less risk for clients to cut back on needed macronutrients. 

## Results: Preliminary data

The study has been the development and collection of preliminary data for Phase I of this treatment program. It was approved by the institutional review board of the University of California, San Diego, and all participants gave written informed consent to participate.

Inclusion criteria include persons who exhibit current symptoms of anorexia nervosa, both restrictor and binge and/or purging subtypes; or have had a history of anorexia nervosa. The treatment program included all genders, ages 18 years and above.

This paper reports on data from 37 clients (36 females and one male;﻿ mean age = 23.2, range = 18-47; mean illness duration = 8.5 years, range = 1-40 years; mean baseline BMI = 18.3 kg/m^2^, range = 13.8-22 kg/m^2^) and 60 supports who completed acceptability and liking ratings after the five-day treatment. Diagnoses were confirmed by using the Structured Clinical Interview for the Diagnostic and Statistical Manual of Mental Disorders, DSM-5 (SCID). Pretesting occurred the weekend prior to the five-day treatment, and post-testing followed on Friday evening or Saturday after treatment. In addition to a battery of standardized tests measuring traits, eating disorder symptoms and weight, daily qualitative information was gathered on what helped the most, what helped the least and what was their primary “take home” message at the end of each day and the end of the week.

The clients and supports are from 17 states and 4 countries, primarily the USA (47 clients). One client and her mother stepped out of treatment after one day due to a family member’s medical emergency, resulting in a two percent (*n* = 1 patient, 1 support) attrition rate in our study, which is lower than around the 30–40 % drop-out rate reported from other treatment studies, which is most probably due to the very short time span of the program [77].

**Fig. 5:**
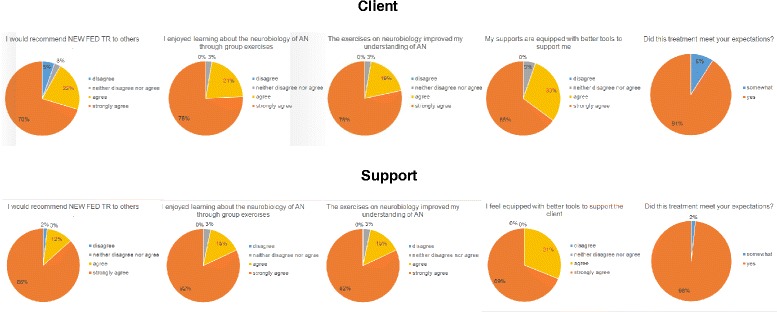
Qualitative Data on the Acceptability of Neurobiological Information from anorexia nervosa Adult Clients and Supports

Preliminary qualitative data indicate good acceptability of the neurobiological information presented (see Fig. 5). Ninety-two percent of the clients and 98 % of the supports would recommend NEW FED TR to others. Ninety-seven percent of the clients and 97 % of the supports reported they enjoyed learning about the neurobiology of eating disorders. Ninety-seven percent of the clients and 97 % of the supports reported the neurobiological exercises improved their understanding of anorexia nervosa. Ninety-five percent of the clients reported that they felt their family/friends were equipped with better support tools and 100 % of supports reported feeling equipped with better support tools. Ninety-one percent of the clients and 98 % of the supports reported that this treatment met their expectations.

There was a significant improvement in learning how to “dose” foods when approaching “food as medicine,” using macronutrients to assure balance in meals. Pre- to post-testing changes in nutritional knowledge in the 32 patients with data resulted in pre mean = 60.2 % accuracy (SD = 21.4), post mean = 78.5 % accuracy (SD = 13.5), t(31) = 5.13, p < 0.001. For the 39 supports who completed the nutritional testing, pre mean = 53.7 % accuracy (SD = 17.0), post mean = 70.7 % accuracy (SD = 10.9), t(38) = 6.11, p > 0.001.

Additional qualitative information on what helped the most during the week and what helped the least during the week are representatively reported by having randomly selected five clients’ and five supports’ feedback for each question (see Table 1). Both clients and supports reported strong and encouraging feedback, with few to minimal concerns for change. The qualitative feedback holds caution in that what is true in the moment at post-testing, may not be true in six months to a year. However, the feedback of Phase I provides a basis for clinical validity for treatment content and interactions, approach to food and the neurobiological basis of Phase I.

**Table 1:** Random Selection of Clients’ and Supports’ Comments of What Helped the Most and the Least

NEW FED TR Clients	
What helped the most?	What helped the least?
The way the brain functions. The noisy brain.	Honestly nothing, and I mean that.
Putting together a treatment plan, discovering the internal workings within the brain, and finding new ways to look at and talk about nutrition.	Nothing
Interaction with other families, neuro education, role playing. [Redacted] is always so incredibly helpful. I think 4 families was a good number.	I didn't really like the DBT sessions - didn't find them that helpful
I have never approached recovery this way and I am hoping it's the way that will work for me.	Nothing. Only regret was not having more time to attend.
Family support based - I will be accountable. Intense, quick - packed with evidence based program. Amazing staff. [Redacted]…life changers. The contract is great. Eliminates questions later.	More heads up BEFORE coming to the program about what the meal plan will be like to minimize the big change/transition to the new meal plan.
NEW FED TR Supports	
What helped the most?	What helped the least?
So much useful information to practice and take home.	Honestly, I understand the benefit to you of getting this feedback and I have struggled with these end-of-day questions.
The program approaches the illness with scientific data. Solutions are good. For the first time, I see an end to this illness.	Really can't say there was anything I didn't like it was a great program.
One on one with team. Brain science studies and examples (restaurant).	(blank)
Being educated about the illness, the meal plan as medicine, making room per individualization, being able to share with other clients and families, the individual therapy session.	Suggest to have [redacted] review the study protocol on Sunday during pre-testing. Also, more structure for dinner Sunday night during pretesting.
[Redacted]’s presentation on the brain function for a patient with ED. Learning more about MY daughter, and why she does what she does - also how to manage recovery. The contract.	Down time

## Discussion

The NEW FED TR preliminary results indicate that adult clients with anorexia nervosa and their supports have a strong desire to learn more detailed information about the neurobiology and biological basis of this illness. As one NEW FED TR mother stated, “Understanding the current neurobiology is critical to family/friend supporters - no excuse not to get this understanding in all treatment programs today. And, for me, this deep understanding had to be explained beyond reading a scientific journal article or watching a PowerPoint.”

Over 90 % of the clients and supports reported that their expectations were met through this new treatment approach. The new, neurobiologically based, experiential treatment tools were well received and evaluated with over 95 % of the participants responding that they agreed or strongly agreed that the tools improved their understanding of the illness. In addition, there was an overwhelming response that both clients and supports would recommend the program to others.

## Conclusions

To date there are little to no effective treatments for adults with anorexia nervosa. Neurobiological research has shifted our understanding of anorexia nervosa from primarily an outside-in, to now include an inside-out, brain-based illness that can be severe, enduring and fatal. Treatment needs to reflect, integrate and synthesize neurobiological underpinnings into ongoing, evidence-based approaches. A new, trait-based model for anorexia nervosa is described, which outlines the genetic and neurobiological vulnerabilities that influence eating disorder development over one’s age span. This model outlines a foundation for a new, five-day, 40 hour, treatment for clients with anorexia nervosa and their supports called NEW FED TR. It integrates neurobiological information and tools within evidence-based family-based treatment, dialectical behavioral therapy and enhanced cognitive behavioral therapeutic approaches. Preliminary qualitative findings reveal that participants agreed or strongly agreed that the neurobiological information was helpful and activities augmented ways to integrate new behavioral patterns into the lifestyles of the clients and families. 

## Additional files

Additional file 1: Behavioral Agreement. (PDF 660 kb)
Additional file 2: Nutrition Facts Questionnaire. (PDF 235 kb)
