# Supplementary material for: Applying neurobiology to the treatment of adults with anorexia nervosa
Source: J Eat Disord. 2016 Dec 5;4:31. doi: 10.1186/s40337-016-0119-x (PMC5137219; doi:10.1186/s40337-016-0119-x)
Supplement: Additional file 1: — Behavioral Agreement. (PDF 660 kb) [file 40337_2016_119_MOESM1_ESM.pdf]

Eating Disorder Behavioral Agreement for NEW FED TR
Client, Treatment Team and Support Person(s).
Five Day Treatment

This is a Treatment Agreement (plan) that can apply up to three months from now.

This 5 day family intensive program is time limited with daily group and family interventions.

Date of first day of treatment: ____________________ Date Agreement is valid through: ________________

Written by (Client name): ____

Current Level of Care:

Residential _____ PHP _____ IOP _____ OP _____

Diagnosis 1: ____

Diagnosis 2: ____

Diagnosis 3: ____

Diagnosis 4: ____

Discharge plans that could occur **after** NEW FED TR include: Residential _____PHP ______IOP _____OP _______

No Treatment Available _______

This agreement can be used when: (check all that apply)

☐ I change from one level of care to another ☐ As my treatment plan

☐ Within my current treatment to hold myself accountable with family/friends

Support is critical for my recovery. In an effort to make myself stronger and my treatment more successful, I am willing to enter into the following agreement with the following support person(s):

a. (S1) ____

b. (S2) ____

c. (S3) ____

d. (S4) ____

Client: Please read out loud each section of the information below.

Change any word(s), phrases or sentences to make the statements **true** for you.

I recognize that I can be my own worst enemy. In my desire to become stronger physically, psychologically and interpersonally, I at times sabotage my own efforts in moving forward. While I may want myself to improve, I at times block myself from progress and push others away who want to help me.

My (Client) Strengths:

My own strengths are major tools and traits that serve as my armor to manage this illness. To commit to the daily battle of overcoming ED, I draw upon my following strengths and traits (T):

☐ Determined
☐ Kind
☐ Generous
☐ Motivated
☐ Intelligent (e.g. good grades, work performance, creative)
☐ Reliable
☐ Open to learn/listen
☐ Attention to detail
☐ Anxiety over time (T)
☐ Impulsivity (T)

☐ Hard worker
☐ Open to seek support
☐ Willing to accept support from others
☐ Perform better in unstructured/spontaneous tasks
☐ Perform better in structured tasks
☐ Performance sensitivity: concern with reward/punishment
☐ Able to problem solve
☐ Obsessive (T)
☐ Persistence (T)
☐ Perfectionism (T)

☐ Tend to hold back & intolerant of uncertainty. (HA) (T)
☐ Competitive (T)
☐ See errors over successes (T)
☐ ____________________
☐ ____________________
☐ ____________________

 © All rights reserved 2016

Client's Strengths *as Viewed by Support:*

The Client's strengths are major tools and traits that serve as his or her armor to manage this illness. To commit to the daily battle of overcoming ED, he or she will draw upon the following strengths and traits (T):

- ☐ Determined
- ☐ Kind
- ☐ Generous
- ☐ Motivated
- ☐ Intelligent (e.g. good grades, work performance, creative)
- ☐ Reliable
- ☐ Open to learn/listen
- ☐ Attention to detail
- ☐ Anxiety over time (T)
- ☐ Impulsivity (T)
- ☐ Hard worker
- ☐ Open to seek support
- ☐ Willing to accept support from others
- ☐ Perform better in unstructured/spontaneous tasks
- ☐ Perform better in structured tasks
- ☐ Performance sensitivity: concern with reward/punishment
- ☐ Able to problem solve
- ☐ Obsessive (T)
- ☐ Persistence (T)
- ☐ Perfectionism (T)
- ☐ Tend to hold back & intolerant of uncertainty. (HA) (T)
- ☐ Competitive (T)
- ☐ See errors over successes (T)
- ☐ ________________
- ☐ ________________
- ☐ ________________

Support Person(s) Strengths *As Viewed by Client:*

S-1	S-2	S-3	S-4	
☐	☐	☐	☐	Reflective Listener: Repeats what s/he hears with no judgment
☐	☐	☐	☐	Willing to learn and practice new tools
☐	☐	☐	☐	Models and owns his/her own emotions (e.g., this is scary for me, I am exhausted, I am determined).
☐	☐	☐	☐	Willingness to try/learn
☐	☐	☐	☐	Able to hold the client accountable
☐	☐	☐	☐	Logical
☐	☐	☐	☐	Won't give up
☐	☐	☐	☐	Keeps an open mind
☐	☐	☐	☐	Problem solver
☐	☐	☐	☐	Other ______________________

Support Person(s) Strengths *As Viewed by Support(s):*

S-1	S-2	S-3	S-4	
☐	☐	☐	☐	Reflective Listener: Repeats what s/he hears with no judgment
☐	☐	☐	☐	Willing to learn and practice new tools
☐	☐	☐	☐	Models and owns his/her own emotions (e.g., this is scary for me, I am exhausted, I am determined).
☐	☐	☐	☐	Willingness to try/learn
☐	☐	☐	☐	Able to hold the client accountable
☐	☐	☐	☐	Logical
☐	☐	☐	☐	Won't give up
☐	☐	☐	☐	Keeps an open mind
☐	☐	☐	☐	Problem solver
☐	☐	☐	☐	Other ______________________

My (Client) Limitations:

I am not very good at the following and need help:

- ☐ Asking for help/support
- ☐ Making and trusting decisions
- ☐ Accepting support from others when I'm about to do or am already doing an ED behavior
- ☐ Obsessive/Compulsive (T)
- ☐ Cognitive Inflexibility (T)
- ☐ Determination
- ☐ Motivation
- ☐ High concern for punishment
- ☐ Competitive (T)
- ☐ ________________
- ☐ Planning ahead
- ☐ Communicating my distress
- ☐ Perfectionism (T)
- ☐ Attention to detail
- ☐ Being kind
- ☐ Being reliable
- ☐ Seeing errors over successes
- ☐ Ability to problem solve
- ☐ ________________
- ☐ Anxiety over time (T)
- ☐ Impulsivity (T)
- ☐ Harm Avoidance (T)
- ☐ Using my intelligence
- ☐ Being generous
- ☐ Being open to learn/listen
- ☐ Persistence (T)
- ☐ Openness to seek support
- ☐ ________________

 © All rights reserved 2016

I agree to be honest (Client)

Truth is essential and difficult to face and to live. If I am not honest with myself and others, trust is lost. Treatment and my own journey become compromised with lies and deception, making the journey ineffective. If change toward health and recovery improves, then honesty is the first step. Your honesty this week and the days, weeks and months after NEW FED TR is fundamental.

- ☐ I agree to share the full truth, even if it makes "ED" angry. Truth keeps me accountable and responsible to myself and others.
- ☐ I recognize that honesty is a skill that needs to be practiced.
- ☐ When I am feeling willful and as though I do not want to recover, I agree/can do the following:

____

____

My (Client) Red Flags include:

- ☐ Eating longer than the agreed amount of time (i.e. snacks, meals and fluids).
- ☐ Change in attire (i.e. wearing baggier clothes).
- ☐ More arguments surrounding food/eating/negotiating treatment/recovery.
- ☐ Avoidance of seeking out help and recovery support network.
- ☐ Misdirecting anger and negative emotions onto family and support people.
- ☐ Increase social withdrawal/isolation/avoidance or previously enjoyed activities.
- ☐ Increase in self-criticism; negative body comments.
- ☐ ____
- ☐ ____

I agree to do/not do the following (Supports):

S-1	S-2	S-3	S-4	
☐	☐	☐	☐	Refrain from making comments about body weight or size.
☐	☐	☐	☐	Refrain from endorsing "diets."
☐	☐	☐	☐	Encourage the balanced meal plan as recommended by the dietitian.
☐	☐	☐	☐	Refrain from judging, but rather ask questions by starting with, "Can you help me understand…?" or "I don't understand…"
☐	☐	☐	☐	Offer 2-3 options to help with decisions regarding work, school and activities.
☐	☐	☐	☐	Active Listening: Repeat back what you hear your loved one saying (validate loved one feelings/thoughts) without adding your own judgment. Specify:__________________________
☐	☐	☐	☐	Other things that are bothersome to the client:

____

____

 © All rights reserved 2016

Background Brain Information

Brain research is finding that those who have eating disorders may experience negative thoughts or loss of control and emotional reactions when eating. There are areas of the brain that appear to be over and under firing or malfunctioning compared to those without eating disorders. Eating healthy portions is very difficult.

Until new brain and biological treatments are developed, you will have to compensate for the brain malfunctions by having others help you with the body shape distress or lack of control over eating and movement. In eating disorders, due to brain response, you may be eating with varying degrees of "blindness." Your brain may not give clear messages for hunger/fullness, taste and satisfaction and/or it may fire acute distress when you eat (with AN and BN) and if you don't eat (with BED). Just as someone may need to assist you if you could not see well, you may need others to help you "walk through eating." Hence, finding healthy ways to compensate for malfunctioning brain signals is necessary, such as planning ahead your meals in detail and having others eat alongside you, whether in person or through text.

Commit to Living my Life with Purpose:

Finding fulfillment outside of ED: This goal is the foundation for all other goals. Please identify something which is a personally fulfilling aspiration that you will commit to fulfilling for the next three months. Identify the action through which you gain fulfillment and purpose. What actions might you take to fulfill a sense of purpose?

Goal: Commit to Living my Life with Purpose (Three Month Goal vs. This Day Goal):

Client: Please read out loud each section of the information below.

Change any word(s), phrases or sentences to make the statements **true** for you.

Life can be more purposeful when you live true to your own skills. And, when you share your skills with others, your purpose is made more complete. Identifying your skills is the first part of living with purpose. The second is to act on them, even if you feel awkward or only see what you do wrong. Sharing your skills creates strength within.

For some with eating disorders, it is hard to look into the future and feel a sense of confidence in what is the "right" thing to do. It can be overwhelming when facing too many options. Thus, sometimes a person with ED needs to "back into life," one step at a time. To develop purpose beyond an eating disorder, write what is purposeful to you at this time in your life.

The following thing(s) are important to me at this time and doing these things can help me push forward through the pain of recovery:

____

____

In the long term, I would want to accomplish ____
_____________________________________by (date:)_______________to achieve purpose.

In the next three months, to give myself a sense of meaning, I would like to ____________________
____

Some examples may include:
-Pursuing higher education or fulfilling employment
-Going out of your way to offer a kind note or comment to another person on a daily basis
-Taking a class on a topic which interests you

 © All rights reserved 2016

What ED changes do I (the client) want to make?

The goals in RED impact ED behaviors and allow you to live purposeful days, weeks, and months. Now that you have identified something purposeful to move toward, please review the goals below and rank them based on importance in changing the ED behaviors in your life. If there is a goal that is not listed, please write it in where listed as "other" and include it in the ranking. 1 = most important to change at this time, 4 or 5 = less focus given to change at this time.

Your choices will be the focus or goal(s) with your support(s) and treatment team this week and for the weeks after this program over the next three months. The treatment team will teach you tools to help you and your support(s) achieve these goals. Our treatment goal is to work WITH you to grow in strength, with the help of your support(s), to live more fully and with purpose. In three months, you may choose to update your agreement with your support(s) and treatment team to keep "on track" in your recovery.

Goals for self-growth and strength to fulfill your purpose:

Please rank each goal below by putting a number in the boxes from 1 (most important) to 4 or 5 (least important)

- ☐ **Commit to my Meal Plan** **pg. 6**
- ☐ **Commit to a Healthy Movement Plan** **pg. 7**
- ☐ **Commit to Reducing Eating Disorder and other Self-Destructive Behaviors** **pg. 8**
- ☐ **Commit to Offering Interpersonal Respect** **pg. 9**
- ☐ **Commit to Other**____ **pg. 9**

Planning is key to working through eating disorder symptoms.

Each goal has objectives listed below it. Objectives are plans of what actions you can take to make the goal become a reality. You may choose one of the objectives listed, several, all or none. If you identify other ways to act on your goal, write it in so both you, your support(s) and treatment team are on the "same page" on how you plan to realize each goal.

Making a Repair:

A repair in eating disorder treatment is holding yourself accountable when you do an ED behavior. An eating disorder behavior is bound to occur. When that happens, move on by making a repair and then act on what you have identified as purposeful. It is important to plan what repairs to make, or it is easy to develop rituals around the eating disorder behaviors instead of new rituals around the new healthy and purposeful actions you are identifying. A repair could also be in a form that transforms the destructive/harmful actions into an action that strengthens yourself and others (e.g. eating and holding it down).

 © All rights reserved 2016

☐ Goal: Commit to my Meal Plan

Client: Please read out loud each section of the information below.

Change any word(s), phrases or sentences in order to make the statements **true** for you.

Food is medicine. As the first round of natural and necessary medical restoration, I need to eat breakfast, lunch, dinner and identified snacks each day, as prescribed, as the foundation for physical strength. I realize that I may have a mental reaction to certain foods that increase my emotional irritability and distort my thinking. My thoughts may become "noisy" and negative and it is hard for me to interact with others. These thoughts can keep me from eating or relentlessly demand for me to eat more, AND yet I need to… (√ only what you choose to do)

Note: you may choose to complete this goal with your dietitian.

I understand and commit to my current meal plan which is: (must be updated as new meal plans are given)

Breakfast:______Carbs,_______Pros,_______EFs,_______Veggies,_______Fluids
Snack 1:______Carbs,________Pros,________EFs,_______Veggies,________Fluids
Lunch:______Carbs,________Pros,________EFs,_______ Veggies,_______Fluids
Snack 2:______Carbs,________Pros,________EFs,_______Veggies,________Fluids
Dinner:______Carbs,_______Pros,________EFs,________Veggies,_______Fluids
Snack 3:______Carbs,________Pros,________EFs,_______Veggies,________Fluids

Client Objectives	Support Person(s) Objectives
☐ Plan meals with ED dietitian<br>☐ Pre-pack lunch_____daily, or every_____days<br>☐ Pre-pack dinner_____daily, or every_____days<br>☐ Identify who will help me prepare my meals/snacks:<br>☐ Myself ☐ Myself with __________________<br>☐ Family/support person __________________<br>☐ Eat 100% of (#)_____meals and____(#) snacks daily (based on treatment team's recommendation)<br>☐ Finish all meals within_____minutes<br>☐ I agree to not negotiate or change the meals<br>☐ I will include identified drinks such as liquid yogurt, probiotic drinks, milk, Gatorade, juice, etc. as part of meal plan.<br>☐ Drink_____oz. of water per day.<br>☐ Be with someone_____minutes following the meal. Name:________________________<br>☐ Create alarms for meals/snacks and other reminders<br>☐ Schedule times for meals/snacks/fluids<br>☐ My meal plan will be monitored by _________________<br>☐ ____<br>☐ ____	S-1 S-2 S-3 S-4<br>☐ ☐ ☐ ☐ I know my loved one's meal plan. If no: Please get a copy from the dietary team.<br>☐ ☐ ☐ ☐ I will provide my loved one with 2-3 choices to eat, vs. "what do you want to eat."<br>☐ ☐ ☐ ☐ Help my loved one come up with a "safety" or "go to" meal for when s/he is struggling.<br>☐ ☐ ☐ ☐ Eat with my loved one during ____________ meals.<br>☐ ☐ ☐ ☐ Text (ID when)______________________<br>☐ ☐ ☐ ☐ Call my loved one to eat with him/her from afar.<br>☐ ☐ ☐ ☐ Agree for meals to be eaten in_____minutes<br>☐ ☐ ☐ ☐ Plan or Pack loved one's ☐ ☐ ☐ ☐ Breakfast ☐ ☐ ☐ ☐ Lunch ☐ ☐ ☐ ☐ Dinner on<br>S-1, S-2, S-3, S-4: ☐ S ☐ M ☐ T ☐ W ☐ Th ☐ F ☐ Sa<br>☐ ____<br>☐ ____

(Continue on next page)

 © All rights reserved 2016

Repairs: If I do an ED behavior instead of meeting my goal on any given day, my repair will be: (check all that apply)

- ☐ Eat with____the following meals ____
- ☐ Hold myself accountable and call out my ED by telling_____________________about my ED action.
- ☐ Seek more structure by entering into a higher level of care for additional structure and support.
- ☐ Eat or drink missed exchanges.

Supplementing if underweight or binge/over eating:

- ☐ If my body composition is low, and if I eat more than 50% but less than 100%, I will drink 1 Boost/Ensure.
- ☐ If my body composition is low, and if I eat less than 50%, I will drink 2 Boosts/Ensures.
- ☐ If I lose control and eat over my recommended meal plan, I will restore balance by eating my next scheduled meal/snack.
- ☐ Other: ____

 © All rights reserved 2016

☐ Goal: Commit to a Healthy Movement Plan

Client: Please read out loud each section of the information below.
Change any word(s), phrases or sentences to make the statements **true** for you.

The brain orders and organizes itself through movement. Although I acknowledge that my eating disorder might have used movement as a self-destructive behavior before, I commit to using my appropriate, prescribed movement plan to calm myself in times of high anxiety, aid in digestion and tolerance of food consumption, and to disrupt or prevent self-destructive behaviors.

I understand my movement plan is level______________currently which includes ___________________________________

____

(must be updated as new movement plans are given)

Client Objectives	Support Person(s) Objectives
☐ I have an identified movement plan from my treatment team, physician, dietitian or coach<br><br>☐ I agree to do the movement plan and share with my support<br><br>☐ If my movement does not:<br>☐ improve__________by *increasing* or *decreasing* (underline that which is the applicable to you)<br>☐ Or maintain _________<br><br>Then I will agree to the altered meal plan and/or to adjust movement as recommended.	S-1 S-2 S-3 S-4<br>☐☐☐☐ I know my loved one's movement plan, if given permission, and will get a copy from ______________________<br>☐☐☐☐ Identify a part of the movement plan you Would like to do with your loved one<br>Activity ____________________<br>Name days ____________________<br><br>☐☐☐☐ Refrain from making comments about fat, weight, shape or body image.<br><br>☐☐☐☐ Refrain from saying the following about movement: ____________________<br>______________________________

Repairs:

☐ Do movement with ____

☐ Not move for________hours_______days

☐ Move with_________________________________hours_______days

☐ Use "fit band" to walk within an agreed range of steps.

☐ Not move beyond what is required to get to work/classes that day.

☐ Not return to college or work until the medical team reports I am strong enough.

☐ I will not be able to go on trip/vacation to________________________________

☐ I will need increased treatment team structure and enter into a higher level of care.

☐ Need to be hospitalized.

☐ Other: ____

 © All rights reserved 2016

☐ **Goal: Commit to Reducing ED and Self-Destructive Behaviors**

Client: Please read out loud each section of the information below.
Change any word(s), phrases or sentences to make the statements **true** for you.

Ed behaviors: I engage in the following self-destructive behavior(s) when my distress or anxiety becomes overwhelming and uncontrollable, I calm myself by turning to (check all that apply):
I commit to reduce the following ED behaviors:

	Eating Disorder Behaviors:	Reduce from:
☐	Self-induced vomiting	_______________to________________X per week
☐	Laxative abuse	_______________to________________X per week
☐	Fasting or restricting	_______________to________________X per week
☐	Diuretics	_______________to________________X per week
☐	Hiding food	_______________to________________X per week
☐	Binge Eat	_______________to________________X per week
☐	Weighing myself_____x per week or_____x per month	_______________to________________X per week
☐	Isolating	_______________to________________X per week
☐	Body checking	_______________to________________X per week
☐	Alcohol (binge drinking) or substance abuse	_______________to________________X per week
☐	Cutting or other self-mutilating behaviors	_______________to________________X per week
☐	Excessive exercise (beyond recommendations)	_______________to________________X per week
☐	Other: ________________ ______________________	_______________to________________X per week

I acknowledge that the temporary relief that I feel from the above self-destructive behavior(s) increases my desire to repeat the self-destructive behavior(s). Drawing upon the structure of treatment and support of family and friends, I commit to use the following tools to bypass these self-destructive behavior(s), forcing myself to manage the distress or seek help to interrupt the urge. I cannot always bypass or interrupt my self-destructive behavior(s) on my own and will rely on my Support(s) to use the above responses to help me bypass these urges or interrupt them in the moment.

I understand that I can use the following tool (page 11) to help interrupt or prevent the ED behaviors:

____

____

____

 © All rights reserved 2016

It would help me if my supports would use the following tools to help me interrupt or prevent my ED behaviors:

___

___

___

Client Objectives	Support Person(s) Objectives
☐ Seek medical care if my self-destructive (ED) behaviors are life threatening<br>☐ Agree to have my weight monitored (if relevant) by ____________________<br>☐ Agree to have my vitals monitored (if relevant) by ____________________<br>☐ Use the tools listed above to interrupt any of my self-destructive (ED) behaviors<br>Use the tools above to prevent any of my chosen self-destructive (ED) behaviors from occurring	S-1 S-2 S-3 S-4<br>☐☐☐☐ I know my loved one's self-destructive behaviors<br>☐☐☐☐ Use the tools listed above to interrupt my loved ones ED behaviors<br>☐☐☐☐ Use the tools listed above to prevent my loved ones ED behaviors<br>☐☐☐☐ If a medical emergency occurs, I understand I must immediately take my loved one to the nearest Urgent Care or Emergency Room.

Repairs if I do a self-destructive (ED) beahvior:

If I hurt myself, I am responsible for my actions and need to (check all that apply):

☐ Share the ED behavior(s) with a support to hold myself accountable. Name______
☐ ____ Agree to increase support during
☐ ____time/day.
☐ If I self-induce vomiting, drink something to replace potassium, such as pure cranberry juice or Gatorade.
If turning to these destructive behaviors more than 2 times a week, I understand more structure is needed. This may include IOP or PHP daily treatment, or more support people at home to intervene.
☐ I will not be able to go back to college/stay in college if I do not stay within the limit identified with the treatment team and/or supports.
☐ If I am over 21 years of age, and I want to drink alcohol responsibly (and has been medically approved), I have met with my dietitian to identify a consumption plan that will ensure health.
☐ I agree to drink alcohol within the limit identified.
☐ I agree to not drink alcohol due to triggers it has on my behavior.
☐ I agree that when I isolate, I will regroup and____________________with____________________.
☐ I agree that, if I body check, I will say ten nice things about myself to______________________________.
☐ I agree that, if partaking in self-harm, I will seek proper medical attention or apply first aid skills.
☐ I agree to tell my support(s),__________________, if I am hiding food and to repair by_______________.
☐ If I have restricted, I agree to repair with____.
☐ After excessive exercise, I agree to____.
☐ Other ____

 © All rights reserved 2016

☐ **Goal: Commit to Offering Interpersonal Respect**

Client: Please read out loud each section of the information below.
Change any word(s), phrases or sentences to make the statements **true** for you.

Interpersonal respect can sometimes be difficult to manage while in the midst of eating disorder noise and anxiety. Therefore, I commit to offer interpersonal respect as often as I am able, with the understanding that, if I am unable to do so, I can take responsibility and offer a repair for my actions.

My Understanding of this goal is: ____
____.

How**:** Check the areas below that you are committed to working on to meet this goal of offering interpersonal respect.

- ☐ I agree to act kindly and with respect to others in the program and outside treatment, at work or school. While I may not yet treat myself with the respect that my body needs, I want myself to practice respect and kindness to others, and in the process, I hope it reflects back onto myself and that I may begin to tolerate or even like myself. I understand that respect means speaking the truth, even if it is not the information I think others may want to hear.
- ☐ I agree to take responsibility for my behavior, draw upon humility and offer a repair, owning my behavior to the person and the group.
- ☐ Recognize my disrespectful action and apologize to the person(s).
- ☐ Other____

I demonstrate interpersonal respect when I do ____

☐ **Additional Goal Not Listed Above**

Describe the goal: ____

Client Objectives	Support Person(s) Objectives
☐ ____________________	S-1 S-2 S-3 S-4<br>☐☐☐☐ ____________________
☐ ____________________	☐☐☐☐ ____________________
☐ ____________________	☐☐☐☐ ____________________
☐ ____________________	☐☐☐☐ ____________________
☐ ____________________	☐☐☐☐ ____________________
	☐☐☐☐ ____________________

What would you be doing differently in three months if you achieved this goal?

____
____
____

 © All rights reserved 2016

Tools to help me accomplish the above goals and objectives (Check which tool(s) you can and want to use):

Client: I Will Practice These Tools

☐	Delay ED behaviors by doing ____________________
☐	Offer support to another person via text, phone or interaction, or spend time with a favorite pet
☐	Deep breathe – inhale 4 seconds, exhale 4 seconds
☐	Plan the next meal/activity, etc.
☐	Distraction
☐	Ground myself to this moment. ID: 2 things I see; 2 things I hear; 2 things I can touch; 2 things I smell
☐	Walk Tall
☐	Wise mind: Apply the dialectic of "I think_______& I feel_______"
☐	Move for 5-10 minutes
☐	Actively participate in a conversation
☐	Hold myself accountable by telling ______________
☐	Stop, reboot, reroute with your support(s)
☐	Reframe my negative desire to an opposite action
☐	WW_____D; or WWID4 ____ (Then just do it)
☐	"Hold the line" (the non-negotiable issue)
☐	Radically accept the action or situation in this moment.
☐	Actively listen
☐	Ask for help
☐	Use pre-meal routine of deep breathing and post-meal of walk 5 minutes (or set amount)
☐	Act with intention
☐	Earphones with music
☐	Medication
☐	Self Critique: (what would I do the same?, What would I do differently? How would I do this?)
☐	Using "I" statements
☐	Planning ahead with sturcture
☐	Use two or three options instead of open ended questions
☐	Restorative yoga
☐	Other: ________________________________

Support(s): I Will Practice These Tools

S-1	S-2	S-3	S-4	
☐	☐	☐	☐	Help loved one delay ED behaviors by doing ____________________
☐	☐	☐	☐	Offer support via text, phone or interaction
☐	☐	☐	☐	Deep breathe with loved one - inhale 4 seconds, exhale 4 seconds
☐	☐	☐	☐	Distract loved one
☐	☐	☐	☐	Help loved one plan ahead next meal/action
☐	☐	☐	☐	Help loved one ground her/himself to this moment via ID: 2 things you see; 2 things you hear; 2 things you can touch; 2 things you smell
☐	☐	☐	☐	Walk Tall
☐	☐	☐	☐	Wise mind: Applying the dialectic of "I think_______& I feel_______"
☐	☐	☐	☐	Move for 5 minutes
☐	☐	☐	☐	Actively participate in a conversation
☐	☐	☐	☐	Hold myself accountable by telling ______________________
☐	☐	☐	☐	Stop, reboot, reroute my loved one
☐	☐	☐	☐	Do pre-meal routine of deep breathing and post meal of walk 5 minutes (or set amount) with your loved one
☐	☐	☐	☐	Actively listen (reflect back what you heard)
☐	☐	☐	☐	"Hold the line" (the non-negotiable area identifed together)
☐	☐	☐	☐	Self Critique: (what would I do the same?, What would I do differently? How would I do this?)
☐	☐	☐	☐	Using "I" statements
☐	☐	☐	☐	"Walking along beside"
☐	☐	☐	☐	Planning ahead with sturcture
☐	☐	☐	☐	Use two or three options instead of open ended questions
☐	☐	☐	☐	Other: ______________________

 © All rights reserved 2016

Trait Continuum from Destructive to Productive Behaviors

Please check the traits that you identified under strengths and limitations on pages 1 and 2. Place an X on the continuum that best describes the expression of your trait over the past month. How have you expressed these traits in a productive way before your ED? Describe a minimum of one productive and one destructive expression of each of your traits in the boxes below.

Destructive Expression of Trait *Productive Expression of Trait*

☐ **Perfectionism**

-2 -1 0 1 2

☐ **Anxiety over time**

-2 -1 0 1 2

☐ **Obsessive**

-2 -1 0 1 2

☐ **Attention to Detail vs Larger Picture**

-2 -1 0 1 2

☐ **Difficulty shifting sets** (difficulty shifting my thoughts from one topic to another)

-2 -1 0 1 2

☐ **Persistent**

-2 -1 0 1 2

☐ **Competitive**

-2 -1 0 1 2

 © All rights reserved 2016

Destructive Expression of Trait *Productive Expression of Trait*

☐ **Difficulty making and trusting decisions** (concern with making the "right" decision)

-2	-1	0	1	2

☐ **Harm Avoidance (with three sub-areas)**

Intolerant of Uncertainty (or the unknowns in life)

-2	-1	0	1	2

Inhibition (hold back when eating, complex activities, faced with too many options all at once)

-2	-1	0	1	2

Inflexibility (for example: I prefer structure)

-2	-1	0	1	2

Interoceptive Awareness: Place an X on the continuum in a location that best describes your ability to experience the following, in general, over the past month.

Hunger

-2	-1	0	1	2
No Sensation				Strong Sensation

Comments: ____________________

Taste

-2	-1	0	1	2
No Sensation (No Flavors)				Strong Sensation (All Flavors)

Comments: ____________________

Satiety

-2	-1	0	1	2
No Sensation				Strong Sensation

Comments: ____________________

Pain Tolerance

-2	-1	0	1	2
No Sensation (High Tolerance)				Strong Sensation (Low Tolerance)

Comments: ____________________

 © All rights reserved 2016

My Discharge Plans:

Discharge plans **after** NEW FED TR look like: Residential______PHP______IOP______OP________RD_______

Frequency of services: ________________________________

No Formal Treatment Available—will draw upon Support (s): 1___, 2___, 3___, 4___

How do <u>you</u> (client) want to use this agreement after NEW FED TR week? (Check all that apply)

- [ ] I will use and hold myself accountable for the next three months.
- [ ] I ask The Center to send a copy to my therapist, email or fax:___________________________________I
- [ ] ask The Center send a copy to my dietitian, email or fax:_______________________________________I
- [ ] ask The Center send a copy to my physician, email or fax:______________________________________I
- [ ] want my supports to use this over the next three months to help hold me accountable (copy given to each support).
- [ ] I will give to the agreement myself to my therapist______my dietitian_____my physician ______
- [ ] I do not plan to use this agreement beyond this treatment week.
- [ ] As I transition from one Level of Care to another
- [ ] As my treatment plan
- [ ] Within current treatment to hold myself accountable with my family and friends
- [ ] Other____

 © All rights reserved 2016

Making my commitment:

By signing this agreement, I am committing to myself, my support(s) and the treatment team(s) the goals and actions I have outlined. I understand that when I "stumble," I move forward by making a repair.

Repairs are as important as the goals. By signing below, I am (and my support(s) is/are) committing to this agreement to guide us forward over the next three months (Date:_______________to______________).

I understand this agreement will be in both my treatment chart at The Center and sent to any treatment team (clinician, dietician and physician) outside The Center.

Signatures: Last day of Treatment:

Signatures:	Last day of Treatment:
______________________________	________________________
Client	Date
______________________________	________________________
Treatment Provider	Date
______________________________	________________________
Dietitian	Date
______________________________	________________________
MD/DO/RN/NP	Date
______________________________	________________________
Friend or Family Member	Date
______________________________	________________________
Friend or Family Member	Date
______________________________	________________________
Friend or Family Member	Date
______________________________	________________________
Friend or Family Member	Date

 © All rights reserved 2016
