# Supplementary material for: Applying neurobiology to the treatment of adults with anorexia nervosa
Source: J Eat Disord. 2016 Dec 5;4:31. doi: 10.1186/s40337-016-0119-x (PMC5137219; doi:10.1186/s40337-016-0119-x)
Supplement: Additional file 2: — Nutrition Facts Questionnaire. (PDF 235 kb) [file 40337_2016_119_MOESM2_ESM.pdf]

Nutrition Facts Questionnaire

Name:_________________________________(print) Date:____________

Pre-test ☐ Post-test ☐

Program
NEW FED TR ☐ PHP ☐ IOP-ED ☐ DBT-IOP ☐ Outpatient ☐

Date you were admitted to current program: _______________

Have you had previous treatment at The Center before the current program of care?
Yes ☐ No ☐

Directions: Please circle any to ALL answers that are true

1. The main function(s) of protein is/are:
a. Build & repair tissue
b. Assist with regulation of body temperature
c. Primary source of fuel for the body
d. Sole source of fuel for the brain

2. The body needs whole grains which provide vitamins, minerals, and fiber. Please choose below any to all that is/are considered a source of whole grains.
a. Apple
b. Saltine crackers
c. Whole wheat pasta
d. White rice

3. Which of the following is(are) a part of a client's movement recommendation at The Center?
a. 10 minute leisure walk
b. 1 hour yoga
c. 10-15 minutes stretching
d. Activities of daily living (ADL's)

4. According to the meal plans provided by the Center's Dietitians, the range for one exchange of carbohydrate is(are):
a. 13-20 grams
b. 15-20 grams
c. 18-23 grams
d. 18-25 grams

5. Which of the following statements is/are true regarding fluid recommendations at The Center?
a. Fluids can contain calories
b. Caffeinated beverages are not recommended
c. Fluid needs can change dependent upon physical activity
d. Everyone should drink up to 8 eight oz. of water daily

Developed by Sonja Stotz, RD, LD; Leah Mako, RD, LD; Kristen Wallace, RD, LD; Alex Lewis, RD, LD
© 2016 The Center for Balanced Living

6. According to the meal plans provided the Center's Dietitians, the range for one exchange of protein is(are):
a. 5-8 grams
b. 5-10 grams
c. 7-10 grams
d. 6-12 grams

7. The main function(s) of fat (endurance fuel) is/are:
a. Build and repair tissue
b. Assist with regulation of body temperature
c. Primary source of fuel for the body
d. Sole source of fuel for the brain

8. Micronutrients are substances that are essential for the growth and development of the human body, but do not provide calories. Which of the following is/are micronutrient(s)?
a. Fiber
b. Vitamins
c. Caffeine
d. Protein

9. Which of the following is/are needed in order to calculate the exchanges for a single food item?
a. Fiber content
b. Total grams of saturated fat
c. Serving size
d. Total grams of added sugar

10. According to the guidelines given by The Center's Dietitians, which of the following would be recommended in an individualized meal plan?
a. Consuming full meal plan in 2-3 meals/snacks per day
b. Consuming full meal plan in 5-6 meals/snacks per day
c. Eating every 60-90 minutes
d. Consuming full meal plan before 7 pm

11. Which of the following contains the highest protein content?
a. 2 eggs
b. ¼ cup almonds
c. 4 oz grilled chicken
d. 4 oz deli meat

12. According to the meal plans provided the Center's Dietitians, the range for one exchange of endurance fuel (fat) is:
a. 3-8 grams
b. 4-9 grams
c. 5-8 grams
d. 5-10 grams

13. Which of the following actions helps your loved one with an eating disorder?
a. Eat with them at meals/snacks
b. Learn their meal plan
c. Tell them they look healthier
d. Ask them what they need for support

Developed by Sonja Stotz, RD, LD; Leah Mako, RD, LD; Kristen Wallace, RD, LD; Alex Lewis, RD, LD
© 2016 The Center for Balanced Living

Please refer to the nutrition label below for the next three (3) questions.

Nutrition Facts

Serving Size 1 cup (228g)
Servings Per Container about 2

Amount Per Serving	
**Calories** 250	Calories from Fat 110
	**% Daily Value***
**Total Fat** 12g	**18%**
Saturated Fat 3g	**15%**
Trans Fat 3g	
**Cholesterol** 30mg	**10%**
**Sodium** 470mg	**20%**
**Total Carbohydrate** 31g	**10%**
Dietary Fiber 0g	**0%**
Sugars 5g	
**Proteins** 5g	
Vitamin A	4%
Vitamin C	2%
Calcium	20%
Iron	4%

* Percent Daily Values are based on a 2,000 calorie diet. Your Daily Values may be higher or lower depending on your calorie needs:

	Calories:	2,000	2,500
Total Fat	Less than	65g	80g
Saturated Fat	Less than	20g	25g
Cholesterol	Less than	300mg	300mg
Sodium	Less than	2,400mg	2,400mg
Total Carbohydrate		300g	375g
Dietary Fiber		25g	30g

For educational purposes only. This label does not meet the labeling requirements described in 21 CFR 101.9.

14. How many carbohydrate exchanges are in one (1) serving of this product?
a. 0
b. 1
c. 2
d. 3

15. How many protein exchanges are in one (1) serving of this product?
a. 0
b. 1
c. 2
d. 3

16. How many fat exchanges are in one (1) serving of this product?
a. 0
b. 1
c. 2
d. 3

17. Which vitamins are fat soluble and should not be supplemented unless recommended by a health professional.
a. A, D, E, K
b. B, C, D, E
c. A, C, D, K
d. A, B, D, E

18. The main function(s) of carbohydrates is/are:
a. Build and repair tissue
b. Assist with regulation of body temperature
c. Primary source of fuel for the body
d. Sole source of fuel for the brain

19. I could explain my meal plan to my support person(s).
a. True
b. False

20. I am able to plan/assemble a meal that meets my “approved” meal plan.
a. True
b. False

21. The following are skills/tools that are helpful for me during meals.

____

____

Developed by Sonja Stotz, RD, LD; Leah Mako, RD, LD; Kristen Wallace, RD, LD; Alex Lewis, RD, LD
© 2016 The Center for Balanced Living

Answers and Scoring Instructions for the Nutrition Facts Questionnaire

The following statements are true:

1. a
2. c
3. a, b, c
4. b
5. a, c
6. c
7. b
8. b
9. c
10. b
11. c
12. c
13. a, b, d
14. c
15. a
16. c
17. a
18. c, d
19. a
20. a
21. ___________________________ any answer.

Scoring:
In order to minimize guessing and maximize knowledge learned,

- every correct answer = +1
- every incorrect answer = -1

For example,

- If one correct answer is checked and there are two correct answers, then the correct answer gets +1 and the unchecked additional correct answer gets a -1
- If an incorrect answer is marked, it is scored as -1

Count the number of +1 and -1's and add up to determine the summative score then add 26 to bring it to a positive bottom line for comparisons in pre and post testing and comparing percentage change.

Maximum score = +52
Lowest score = -21

Developed by Sonja Stotz, RD, LD; Leah Mako, RD, LD; Kristen Wallace, RD, LD; Alex Lewis, RD, LD
© 2016 The Center for Balanced Living
